# Society Meetings

**Published:** 1909-06

**Authors:** 


### SOCIETY MEETINGS.

The Buffalo Academy of Medicine held meetings during the months of April and May, 1909, as follows:

*Section on Surgery.*—Tuesday evening, April 6, 1909 at 8.30 P. M. Program. Symposium on diseases of the gall-bladder. (1) Gastric symptoms and complications of disease of the gall-bladder, Allen A. Jones; (2) Diagnosis and surgical treatment of diseases of the gall-bladder, Martin B. Tinker, Lecturer in Surgery at Cornell University Medical College, Ithaca; (3) complications arising in the surgical treatment of diseases of the gall-bladder, Roswell Park.

*Section on Medicine.*—The regular meeting of this section was held at the Academy Rooms, Public Library Building on Tuesday evening, April 13, 1909, at 8.30 P. M. Program: (1) Report of two cases of brain tumor, with exhibition of specimens, Henry P. Frost, Assistant Superintendent Buffalo State Hospital; (2) Report of three cases of pyloric stenosis in infants, Irving M. Snow; (3) Pneumopericardium, with report of a case, Joseph Burke; (4) Medical work in Paris hospitals, A. L. Benedict.

*Section on Pathology.*—Tuesday evening, April 20, 1909, at 8.30 P. M. Program: The essayist for the evening was Harlow Brooks, of New York city, Assistant Professor of Pathologic Anatomy, University and Bellevue Hospital Medical School. Subject, An anatomical study of pericarditis with its clinical deductions. The discussion was opened by Dr. Charles G. Stockton.

*Section on Obstetrics and Gynecology.*—Tuesday evening, April 27, 1909, at 8.30 P. M. Program: The relative value of accouchement force in general practice with special reference to vaginal cesarean section, Reuben Peterson, Professor of Obstetrics and Gynecology University of Michigan, Gynecologist and Obstetrician to the University Hospital; Fellow American Gynecological Association; Fellow American Academy of Medicine; Member of Chicago Gynecological Society. The paper was illustrated by magic lantern slides. Discussion by Drs. Mann, Frederick, Schroeter, Van Peyma, Hayd, King and others.

*Section on Surgery.*—Tuesday evening, May 4, 1909, at 8.30 P. M. Program: Doctors' mistakes, Geo. W. Gay, Boston, Mass., Senior Attending Surgeon of Boston city Hospital, Ex-President, Massachusetts State Society, Lecturer in Surgery at the Harvard Medical School.

*Section on Medicine.*—Tuesday evening, May 11, 1909, at 8.30 P. M. Program: (1) Report of three cases of pyloric stenosis in infants, Irving M. Snow; (2) Pyelitis, A. E. Woehnert; (3) Splanchnoptosis, A. T. Lytle.

*Section on Pathology.*—Tuesday evening, May 18, 1909, at 8.30 P. M. Program: Studies in experimental nephritis and their bearing on renal changes in man, John J. MacKenzie, Professor of Pathology and Bacteriology University of Toronto.

The annual election of officers for this section for the ensuing year was held at this meeting.

---

THE Rochester Academy of Medicine held meetings during the months of April and May, 1909, at the Hotel Seneca, as follows:

*General Medicine.*—Wednesday, April 7, 1909, at 8.15 P. M. Program: The psychic mechanism of hysterical phenomena as determined by Breuer and Freud, Haydon Rochester; Some remarks on anemia, C. O. Boswell.

Wednesday, May 5. Program: The nervous woman, E. B. Angell; Some remarks on anemia, Charles O. Boswell.

*Surgery.*—Friday, April 16, 1909, at 8.15 P. M. Program: Four kinds of appendicitis, Robert T. Morris, New York.

Wednesday, May 12. Program: Movable kidney as a pathological factor—two unusual cases, Charles R. Barber.

Candidates for resident and non-resident fellowship were elected at this meeting.

*Obstetrics, Gynecology, and Pediatrics.*—Wednesday, April 21, 1909, at 8.15 P. M. Program: Suppurative peritonitis, H. L. St. John (by invitation); An unusual case of vicarious menstruation, L. W. Howk.

*Public Health.*—Wednesday, April 28, 1909, at 8.15 P. M. Program: Present status of vaccine therapy, George W. Ross, University of Toronto.

*Obstetrics, Gynecology, and Pediatrics.*—Wednesday, May 19, at 8.15 P. M. Program: Anteflexion of the uterus, Marion Craig Potter. Discussion opened by F. W. Zimmer.

---

The Medical Society of the County of Niagara, at its annual meeting held at the Imperial Hotel, May 10, 1909, elected the following named officers: president, E. B. Horton; vice-president, L. H. Teeter; secretary-treasurer, J. H. Miller; censors and trustees, A. J. Lawler, one year; W. A. Scott, two years; C. G. Leo-Wolf, three years.

---

The American Medical Association will hold its sixtieth annual meeting at Atlantic City, June 8-11, 1909, under the presidency of Col. William C. Gorgas, M.D., U. S. Army. Railway rates have been established at a fare and a half under the usual conditions.

---

The American Protologic Society will hold its eleventh annual meeting at Atlantic City, N. J., June 7 and 8, 1909. The headquarters and place of meeting are at Haddon Hall. The profession is cordially invited to attend all meetings. Dr. Lewis H. Adler, Jr., of Philadelphia, is the secretary.

THE American Medical Editors Association will hold its fortieth annual meeting at Atlantic City, N. J., June, 1909. The officers are: President, T. D. Crothers, editor, *Quarterly Journal of Inebriety,* Hartford, Conn.; first vice-president, W. A. Young, managing editor, *Canadian Journal of Medicine and Surgery,* Toronto, Ont., Can.; second vice-president, E. W. Taylor, editor, *Boston Medical and Surgical Journal,* Boston, Mass.; secretary-treasurer, Joseph MacDonald, Jr., managing editor of the *American Journal of Surgery,* New York, N. Y. Office of the secretary and treasurer, 92 William St., New York City.

---

THE National Confederation of State Medical Examining and Licensing Boards will hold its nineteenth annual meeting at Atlantic City, Monday, June 7, 1909, in the Park Avenue Hall of the Hotel Marlborough-Blenheim. The president, Dr. T. J. Happel, being ill, the first vice-president, Dr. A. Ravogli, Cincinnati, assumes the duties of the chair. Three sessions will be held, one at 10 o'clock in the morning, one at 2 o'clock in the afternoon, and a final one at 8 o'clock in the evening. The program is:

I. Annual Address, by the Vice-President, A. Ravogli, Cincinnati, Ohio. The Necessity of a Practical Test for Candidates in the State Examinations.

II. Report of the Committee on Standing of Medical Colleges, James A. Duncan, Vice-Chairman, Toledo, Ohio.

III. Symposium on Medical Colleges and Their Equipment. (1) The Need, Methods and Value of Medical College Inspection, N. P. Colwell, Chicago, Ill.; (2) The Standing of Medical Colleges in the Light of State Board Examinations, Geo. H. Simmons, Chicago, Ill.

Discussion opened by Arthur Dean Bevan, Chicago, Ill.; James A. Duncan, Toledo, O.; W. J. Means, Ohio University, Columbus, O.; F. Forchheimer, University, Cincinnati, O.; and Geo. H. Quay, Homeopathic Medical College; R. A. Baker, College Physician and Surgeon; F. C. Wait, Western Reserve University, Cleveland, O.

IV. Report of the Legislative Committee, Edwin B. Harvey, Chairman, Boston, Mass.

V. Report of the Examinations Committee, J. C. Guernsey, Vice-Chairman, Philadelphia, Pa.

VI. Symposium on Examinations: (1) Practical *versus* Theoretical Examinations, W. T. Councilman, Boston, Mass.; (2) How Can Practical Examinations be Conducted and What

Should They Include? Charles A. L. Reed, Cincinnati, Ohio; (3) The Inadequacy of the Written Examination, Edwin B. Harvey, Boston, Mass.; (4) What Branches or Portions of Branches Should be Included in the Written, and What in the Oral Examinations? J. C. Guernsey, Philadelphia, Pa.; (5) Is it to the Best Interests of Students and Medical Examining Boards to have Divided Examinations? William Warren Potter, Buffalo, N. Y.; (6) The Feasibility of Practical Examinations, Augustus Korndoerfer, Philadelphia, Pa.; (7) How Can Practical Examinations be Graded and Recorded? L. F. Bennett, Beloit, Wis.

Discussion, opened by V. C. Vaughan, Ann Arbor, Mich., Herbert L. Northrop and Henry Beates, Philadelphia, Pa.

VII. Report of the Secretary-Treasurer, Murray Galt Motter, Washington, D. C.

VIII. Miscellaneous Business.

IX. Report of the Executive Council, N. R. Coleman, Chairman, Columbus, Ohio.

X. Election of Officers.

XI. Adjournment.

